# Direct non-cyclooxygenase-2 targets of celecoxib and their potential relevance for cancer therapy

**DOI:** 10.1038/sj.bjc.6604049

**Published:** 2007-10-23

**Authors:** A H Schönthal

**Affiliations:** 1 Department of Molecular Microbiology and Immunology, Keck School of Medicine, University of Southern California, Los Angeles, CA, USA

**Keywords:** endoplasmic reticulum (ER) stress, carbonic anhydrases, 3-phosphoinositide-dependent protein kinase-1, cyclooxygenase-2 independence, coxibs

## Abstract

Celecoxib (Celebrex®) was developed as a selective cyclooxygenase-2 (COX-2) inhibitor for the treatment of chronic pain. However, it now appears that this compound harbours additional pharmacologic activities that are entirely independent of its COX-2-inhibitory activity. This review presents the recently emerged direct non-COX-2 targets of celecoxib and their proposed role in mediating this drug's antitumour effects.

Although celecoxib (Celebrex) was developed as a selective cyclooxygenase-2 (COX-2) inhibitor, additional pharmacologic activities have emerged outside of its analgesic activity. For instance, its potency to inhibit COX-2 in the nanomolar range has been eclipsed by its ability to inhibit various isoforms of carbonic anhydrase (CA) at even lower concentrations (see below). Moreover, when added to cells in culture at moderate micromolar concentrations, celecoxib was shown to affect several additional cellular components with roles in cellular proliferation and survival (Grösch *et al*, 2006).

When employed in the micromolar range in cell culture, the molecular mechanism by which celecoxib exerts its myriad of pharmacologic activities has not been fully elucidated. But among the various targets, there are at least two cellular components that seem to be controlled directly by celecoxib and thus appear to represent receptors that are engaged at moderate micromolar concentrations. One of these is 3-phosphoinositide-dependent protein kinase-1 (PDK1) and the other is sarcoplasmic/endoplasmic reticulum (ER) calcium ATPase (SERCA). When studied *in vitro*, inhibition of both PDK1 and SERCA requires substantially higher drug concentrations than are necessary to inhibit either COX-2 or CAs; nonetheless, recent evidence obtained from the use of animal tumour models demonstrates that PDK-1 and SERCA are also affected by celecoxib *in vivo* (see below). Therefore, these non-COX-2 targets of celecoxib should be taken into account when interpreting experimental results obtained from the use of celecoxib.

The above findings indicate that the ‘selective’ inhibitor celecoxib has lost its selectivity over time, simply due to the discovery of additional drug targets. This poses the obvious dilemma that observed drug effects cannot be easily ascribed solely to the inhibition of COX-2, but must be supported by careful controls in order to establish the relevant mechanism of drug action. In other words, even though inhibition of COX-2 may take place, this observation by itself does not establish this particular pharmacologic activity as the underlying mechanism by which celecoxib exerts its antitumour properties. For instance, if certain phenotypic consequences observed with celecoxib were attributed to the inhibition of COX-2 activity alone, one would expect that other COX-2 inhibitors, such as rofecoxib (Vioxx®), valdecoxib (Bextra®) or those traditional non-steroidal anti-inflammatory drugs (NSAIDs) that inhibit both COX-1 and COX-2, should also demonstrate these types of biological activities. This is clearly the case in many inflammatory conditions, where the activities seen with NSAIDs can be attributed to COX-2 inhibition. In addition, chemoprevention of colon cancer is also an established pharmacologic activity where inhibition of COX-2 correlates with the suppression of tumour development (Koehne and Dubois, 2004).

In contrast to the foregoing, the antitumour activities of celecoxib in advanced cancers are not well delineated (Kashfi and Rigas, 2005). On one hand, there is clear evidence that COX-2 is an important player even in advanced tumours, where a constellation of other cellular components, such as activated oncogenes and inactivated tumour suppressors, has usurped growth control and drives the malignant expansion. On the other hand, there are an increasing number of reports indicating that celecoxib does not require the presence of COX-2 in order to exert its antitumour activities (Kashfi and Rigas, 2005; Grösch *et al*, 2006; Schönthal, 2007). Even more striking, it was demonstrated that close structural analogues of celecoxib – devoid of any COX-2 inhibitory activity – were able to potently mimic all antitumour properties of celecoxib investigated so far, not just *in vitro* but also in various xenograft animal tumour models *in vivo* (Song *et al*, 2002; Kulp *et al*, 2004; Schönthal, 2006). In the absence of COX-2 inhibition, the recently emerged non-COX-2 receptors and targets of celecoxib are obvious candidates to mediate these antitumour effects in advanced cancers.

The following summary will primarily focus on those non-COX-2 targets of celecoxib that are able to directly bind to the drug and thus appear to represent additional intracellular drug receptors. Owing to space restrictions, other established COX-2-independent targets of celecoxib will not be discussed in greater detail (Kashfi and Rigas, 2005; Grösch *et al*, 2006). Moreover, the very large body of literature establishing and supporting a central role for COX-2 in the actions of celecoxib will not be presented here; the interested reader is referred to some of the excellent recent reviews in that area (Fürstenberger *et al*, 2006).

## CARBONIC ANHYDRASES AS TARGETS FOR CELECOXIB

More than a dozen different isoforms of CA are known. They are zinc metalloenzymes that catalyse the reversible interconversion of carbon dioxide and bicarbonate and thereby regulate physiological pH and other processes. At least two of them, CAs IX and XII (CAIX and CAXII), are expressed in a wide variety of malignancies and implicated in tumour growth (see details in reference Pastorekova *et al*, 2007). CAIX in particular is linked to poor prognosis in a number of human tumours. Its pronounced induction under hypoxia is thought to support tumour cell growth under such adverse conditions and might contribute to resistance against cytotoxic chemotherapy (Potter and Harris, 2003).

Further evidence of the importance of certain CAs in the oncogenic process comes from investigations of clinically relevant CA inhibitors, most notably, the heterocyclic and aromatic sulphonamides, such as acetazolamide, methazolamide or ethoxzolamide, which are normally used for glaucoma and other medical purposes. Several of these CA inhibitors have demonstrated pronounced antitumour effects in various *in vitro* and *in vivo* models (Supuran *et al*, 2003).

**Figure 1:**
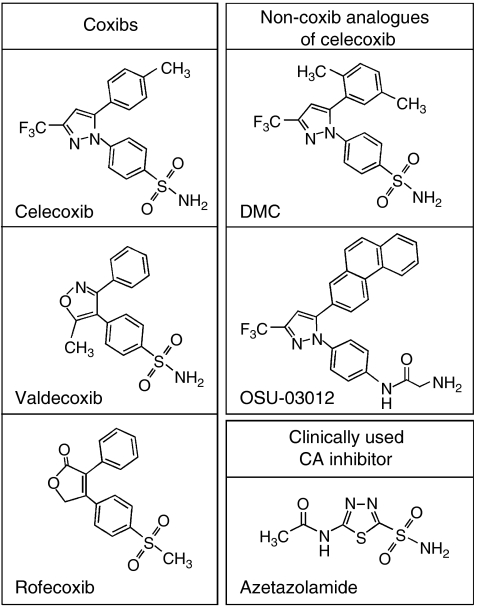
Chemical structures of various anticancer compounds.

Celecoxib, like the prototype CA inhibitor acetazolamide, is structurally characterised by an unsubstituted sulphonamide moiety (Figure 1). Despite this telling chemical similarity, it came as a surprise when it was discovered that celecoxib displayed potent CA inhibitory activity in the low nanomolar range *in vitro* (Knudsen *et al*, 2004; Weber *et al*, 2004). In fact, its IC_50_ towards the tumour-associated CAIX and CAXII enzymes was determined to be 16 and 18 nM, respectively (Di Fiore *et al*, 2006), and thus was more than double as potent than its inhibition of COX-2, where the IC_50_ is 40 nM (Penning *et al*, 1997). Similarly, valdecoxib, a sulphonamide-based coxib as well (Figure 1), was able to inhibit CAIX and CAXII with comparable potency, although its binding mode was entirely different from celecoxib (Di Fiore *et al*, 2006). In contrast, several non-sulphonamide COX inhibitors tested, including rofecoxib, a methylsulphone-type coxib (Figure 1), had no CA inhibitory activity (Knudsen *et al*, 2004; Weber *et al*, 2004). The ability of celecoxib and valdecoxib to inhibit CAs *in vivo* was confirmed in glaucomatous rabbits, where both drugs were able to lower intraocular pressure, suggesting that these agents may have utility in the treatment of this disorder (Weber *et al*, 2004).

The proposed role of CAIX and CAXII in tumour proliferation, progression and chemoresistance begs the question as to whether these enzymes might represent potentially crucial players in the antitumour mechanisms of celecoxib. Unfortunately, at present, there are no data available to support such an assessment.

## PDK1 AS A TARGET FOR CELECOXIB

Much excitement was generated by the finding that celecoxib could bind to and inhibit PDK1 (Arico *et al*, 2002; Kulp *et al*, 2004). PDK1 is a crucial component of cell growth and survival signalling pathways that also involve its upstream regulator PI3K (phosphatitylinositol-3-kinase), its major downstream substrate Akt/PKB (protein kinase B) and the tumour suppressor PTEN (phosphatase and tensin homologue deleted on chromosome 10), which acts as a negative regulator of PI3K/PDK1/Akt signalling. In many tumour cells, especially in those with deleted PTEN, the PI3K/PDK1/Akt axis is chronically activated and contributes to tumour growth and chemoresistance. Considering the master regulatory role of PDK1 in pro-oncogenic pathways, the discovery of its inhibition by celecoxib provided a suitable explanation for the drug's COX-2-independent antitumour effects (Hsu *et al*, 2000; Kulp *et al*, 2004).

In comparison to COX-2 or CA inhibition, significantly higher concentrations of celecoxib are required to inhibit the enzymatic activity of PDK1 *in vitro*, and IC_50_s from 3.5 *μ*M (Arico *et al*, 2002) to 48 *μ*M were reported (Kulp *et al*, 2004). The latter value is more in line with the growth-inhibitory and apoptosis-inducing potential of celecoxib in cell culture, where generally concentrations in the range of 30–100 *μ*M are needed to exert pronounced antiproliferative effects.

Despite several lines of evidence that PDK1 could be a major COX-2-independent receptor of celecoxib, further studies raised some concerns as to the general validity of this model. For instance, in our own studies, we did not detect substantial celecoxib-mediated inhibition of PDK1 (Kardosh *et al*, 2005). Furthermore, celecoxib induced apoptosis with equal efficacy in mouse embryonic fibroblasts lacking both PDK1 alleles as compared to their wild-type counterparts that expressed PDK1 normally (Kardosh *et al*, 2005). Moreover, the downregulation of phosphorylation of the primary PDK1 target Akt, which has been reported in several celecoxib-treated tumour cell lines (Hsu *et al*, 2000; Arico *et al*, 2002; Song *et al*, 2002; Kulp *et al*, 2004), is not consistently observed in all tumour cells (Kern *et al*, 2002; Kardosh *et al*, 2005), even though apoptosis is similarly induced by drug treatment.

Although questions remain as to the precise role of PDK1 in celecoxib-induced antitumour processes, the recognition of PDK1 as a target of celecoxib prompted subsequent structure-activity analysis combined with molecular modelling to generate COX-2 inactive celecoxib derivatives with increased potency towards PDK1, such as OSU-03012 (Figure 1) (Zhu *et al*, 2004). Based on the key role of PDK1 in tumour growth, such streamlined compounds are expected to be useful in cancer therapy.

## SERCA AS A TARGET FOR CELECOXIB

The discovery (Johnson *et al*, 2002) that celecoxib is able to inhibit the sarcoplasmic/ER calcium ATPase (SERCA) set the stage for subsequent studies that eventually established the ER stress response (ESR) as a crucial non-COX-2 target of celecoxib. SERCA is a transmembrane ER protein that maintains the steep gradient of calcium between the cytosol and the ER. Many studies with thapsigargin, a widely used model inhibitor of SERCA, have established that inhibition of this pump results in rapid leakage of calcium into the cytosol; as a result, the ESR is triggered. The primary purpose of the ESR is to alleviate the respective stressful disturbance and restore proper ER homeostasis; however, in the case of intense or persistent ER stress, as appears to be the case in the continued presence of celecoxib, this mechanism will trigger programmed cell death/apoptosis.

A number of papers (Johnson *et al*, 2002; Tanaka *et al*, 2005; Alloza *et al*, 2006; Pyrko *et al*, 2007a) have demonstrated that calcium release from the ER is the most immediate effect of celecoxib treatment and can be detected within seconds of adding the drug to cells in culture. As a consequence, and very similar to what has been established for the model inducer thapsigargin, the typical features of severe ESR can be observed (Tsutsumi *et al*, 2004, 2006; Kim *et al*, 2007; Pyrko *et al*, 2007a). Among these features is the phosphorylation and inactivation of translation initiation factor 2*α* (eIF2*α*), which causes a prominent, yet transient, shutdown of general protein synthesis (Pyrko *et al*, 2007a, 2007b). Exempt from this inhibition of general translation are ESR-specific proteins, such as the chaperone protein GRP78 (glucose-regulated protein with molecular weight 78 kDa) or CHOP/GADD153 (CCAAT/enhancer binding protein homologous transcription factor), an important transcription factor that is involved in mediating ESR-induced apoptosis. Both of these proteins are strongly induced by celecoxib (Tsutsumi *et al*, 2004, 2006; Pyrko *et al*, 2007a, 2007b).

The substantial ESR-mediated downregulation of general translation by celecoxib might have considerable implications for the interpretation of some of the other reported COX-2-independent effects of this drug. For example, celecoxib treatment of cells in culture has been shown to result in cell cycle arrest (Grösch *et al*, 2001; Kardosh *et al*, 2004). This appears to be due to the downregulation of various cyclin proteins, which are the essential subunits of cyclin-dependent kinases (CDKs) that constitute the cell cycle engine and drives the cells through the cell cycle. Loss of CDK activity prevents the phosphorylation of CDK target proteins, most prominent among them the retinoblastoma (Rb) tumour suppressor protein, which remains hypophosphorylated and thus enforces the restriction point and concomitant growth arrest in G1. It is quite tempting to conjecture that all of these known effects of celecoxib, that is, downregulation of cyclins, inhibition of CDK activity, loss of Rb phosphorylation, and ensuing cell cycle arrest could result from the drug's inhibition of SERCA: inhibition of this pump generates increased cytosolic calcium levels, which trigger severe ER stress and attenuate general protein synthesis; as a consequence, short-lived proteins, such as cyclin D, disappear quickly and cannot be replenished. Without cyclin D, the essential G1 CDKs become inactive and incapable of phosphorylating their major substrate Rb protein; as a result, hypophosphorylated Rb remains active and prevents cells from progressing towards S phase. Thus, in this scenario, the effects of celecoxib on SERCA and ER stress would suffice to explain its seemingly unrelated effects on the cell cycle machinery.

Activation of the ESR has also been detected in tumour tissue from celecoxib-treated animals, clearly demonstrating that these *in vitro* effects of the drug also take place *in vivo* (Tsutsumi *et al*, 2004; Pyrko *et al*, 2007a). This latter observation is particularly important in view of earlier scepticism as to the relevance of *in vitro* findings with celecoxib (Williams *et al*, 2000). As many COX-2-independent effects of celecoxib, including the stimulation of ESR, only take place at elevated concentrations, that is in the range of 10–100 *μ*M *in vitro*, it was questioned whether these results might have relevance *in vivo*, where drug concentrations generally are below 5 *μ*M. The detection of ER stress in tumour tissue from celecoxib-treated animals clearly indicated that the *in vitro* observations have *in vivo* relevance – although the conundrum of the concentration differential between *in vitro* and *in vivo* conditions remains, and the potential role of additional, still unknown targets of celecoxib cannot be excluded.

Intriguingly, ESR-inducing activity is also displayed by 2,5-dimethyl-celecoxib (DMC) (Pyrko *et al*, 2007a), a well-studied structural analogue of celecoxib that lacks COX-2 inhibitory function (Figure 1). In fact, DMC has been shown to faithfully mimic – at increased potency – each and every one of celecoxib's non-COX-2 effects investigated so far *in vitro* and *in vivo* (Kulp *et al*, 2004; Schönthal, 2006), further substantiating the presence of multiple activities within the celecoxib molecule.

## CONCLUSIONS

Celecoxib is unique among the coxibs and traditional NSAIDs, because this particular drug displays the greatest potency to induce apoptotic cell death. This activity does not correlate with the inhibition of COX-2, but is congruent with its unique ability to inhibit the non-COX-2 targets PDK1 and SERCA at moderate micromolar concentrations. The inhibition of SERCA constitutes a very rapid drug effect, as increased concentrations of cytosolic calcium levels can be measured within seconds after the addition of celecoxib to intact cells. Significantly, inhibition of these non-COX-2 targets by celecoxib also seems to occur in animal tumour models *in vivo*, arguing against earlier concerns that such effects might be artefacts of the high drug concentrations used in cell culture systems *in vitro*. Additionally, celecoxib is able to inhibit the tumour-associated CAs IX and XII at nanomolar concentrations that are below those required for inhibition of its original target, COX-2.

Although it shall remain undisputed that the inhibition of COX-2, as exerted by celecoxib, has clinically relevant antineoplastic applications, it is also apparent that the celecoxib molecule harbours additional activities that may exert antitumour functions independent of the COX-2 inhibitory activity. It is therefore important to consider the entirety of these multifaceted effects when interpreting data obtained from the experimental use of celecoxib.
